# The first study on population knowledge and attitudes regarding prevention, diagnostic methods, treatment, and recovery aspects related to *Helicobacter pylori* infection in Bosnia and Herzegovina

**DOI:** 10.3389/fpubh.2025.1498407

**Published:** 2025-03-18

**Authors:** Nejra Bektas, Larisa Besic, Aida Kulo Cesic, Damir Marjanovic, Jasminka Prguda-Mujic

**Affiliations:** ^1^Department of Genetics and Bioengineering, International Burch University, Sarajevo, Bosnia and Herzegovina; ^2^Department of Pharmacology, Clinical Pharmacology and Toxicology, Medical Faculty, University of Sarajevo, Sarajevo, Bosnia and Herzegovina; ^3^Institute for Anthropological Research, Zagreb, Croatia; ^4^Health Institute Biomedical Diagnostics and Research Medicover Diagnostics, Sarajevo, Bosnia and Herzegovina

**Keywords:** *Helicobacter pylori*, knowledge, population, infection, survey

## Abstract

The aim of this pioneering study was to examine the knowledge and attitudes regarding prevention, diagnostic methods, treatment, and recovery aspects related to *Helicobacter pylori* infection within the general population of Bosnia and Herzegovina (B&H). Study was conducted using the previously designed questionnaire, adapted for the B&H population. The research enrolled 1,031 participants, of whom 58.49% answered predominantly correctly to questions regarding *Helicobacter pylori* infectivity. Of all participants, 36.18% underwent screening, with 65.95% testing positive, and of those, 93.90% received treatment, mainly antibiotics (92.64%). Of those treated, 74.46% were re-tested and 30.23% of them had relapsed infection. Furthermore, the study identified lower infection rate in younger participants and, contraversaly, in participants with the history of long-term (lasting for more than a year) alcohol consumption, who were also shown to report symptoms' improvement post-treatment. Overall, B&H population demonstrated good knowledge toward *Helicobacter pylori* infection, with higher levels of knowledge in women, highly educated, or screened for *H. pylori*. Notably, participants expressed strong support for national *Helicobacter pylori* screening and thus underscored the importance of planning it in the public health initiatives in B&H. Also, due to the high relapsed infection rate, further effort needs to be directed toward education of risk groups i.e., older age groups, and community on effective measures for *Helicobacter pylori* prevention and treatment.

## Introduction

The gram-negative bacterium *Helicobacter pylori* (*H. pylori*) impacts an estimated 4.4 billion people worldwide, marking it a widespread and significant concern in global health (1, 2), especially after being classified as class I carcinogen associated with the onset of gastric cancer (GC) (3). It can also lead to chronic gastritis, peptic ulcers, and mucosa-associated lymphoid tissue lymphoma (1).

Its prevalence shows variation not only across different countries but also within various regions of the same country (4). The infection tends to be more prevalent among people with lower socioeconomic status and in developing nations (5). Hooi et al. even claimed that *H. pylori* infection is a global one, and it is not resolving spontaneously (6).

*H. pylori* is a helix-shaped, microaerophilic, flagellated bacteria that can shape from spiral to coccoid form. These forms seem to help its survival in the host gastric microenvironment, with the spiral form that enables its successful motility, whereas the coccoid form provides its ability to colonize the mucus layer of the gastric epithelium, enhancing its further invasiveness (7). The precise mode of transmission is unclear. However, new infections are thought to occur because of direct human-to-human transmission, via fecal-oral, gastric-oral, oral-oral route. Moreover, in developing countries, *H. pylori* infection rates increase rapidly within the first 5 years of life and remain consistently high thereafter, suggesting that the bacterium is primarily acquired in early childhood (8–11).

The major challenge in *H. pylori* eradication is its resistance to antibiotics (12). It is primarily attributed to chromosomal mutations, but additional mechanisms such as efflux pump activity, alterations in membrane permeability, and biofilm formation also contribute to resistance (13–15).

A recent comprehensive review and meta-analysis showed alarming levels of primary and secondary resistance rates to clarithromycin, metronidazole, and levofloxacin, surpassing 15% across all World Health Organization (WHO) regions (16). Moreover, most treatments are administered without prior assessment of antibiotic resistance leading to decreased success of available therapies and increased rate of *H. pylori* recurrence, a crucial factor impacting its prevalence. The study of the global recurrence rate indicated an escalating annual *H. pylori* recurrence rate over the decades, rising from 3.9% in the 1990s to 4.8% in the 2010s, potentially contributing to an increase in the number of infected individuals (17). Notably, a study conducted by Zhao et al. showed that European nations exhibited the highest recurrence rates among the regions analyzed (18).

Despite concerted efforts to address *H. pylori* infection, global public awareness of this issue remains notably low. Moreover, studies conducted among the general population (19) and students (20) have consistently shown insufficient knowledge about *H. pylori*. Another study showed a lack of knowledge about the established diagnostic and treatment recommendations for *H. pylori* infection among general practitioners (21). This suggests an urgent need for enhanced education within the realm of continuing medical education, aiming to improve both physicians' and patients' awareness and knowledge concerning *H. pylori* infection.

The data concerning the incidence of *H. pylori* infection in Bosnia and Herzegovina (B&H) are lacking, and of GC are old and scarce. Studies reported the GC incidence of 1.26% in 2005 (22) and of 2.21% in 2009 (23). According to the more recent data published by the International Agency of Research on Cancer (IARC) in 2018, the incidence of gastric adenocarcinoma (GAC) in B&H has increased to 10.1%, with the mortality rate of 7.4% (24). Nonetheless, the general understanding of *H. pylori* prevention and treatment among the population of B&H has never been investigated.

Therefore, the aim of this study was to conduct a comprehensive national survey focusing on the knowledge and attitudes regarding prevention, diagnostic methods, treatment, and recovery aspects related to *H. pylori* infection in the B&H population.

## Materials and methods

### Study design and population

The study was an online survey-based study, conducted between the 8th June 2024 and 8th September 2024. It engaged citizens of B&H aged 18 and above. The minimum sample size was calculated to be 384, as determined using the formula *n* = *N* × *Z*^2^ × *p* × (1 − *p*)÷(*N* − 1) × *d*^2^+*Z*^2^ × *p* × (1 − *p*) (25), in which the confidence level (*Z*) was 95% or 1.96, the maximum variability (*p*) was 0.5 and margin of error (d) was 5% or 0.05. Prior to participation, participants were asked for verbal consent which was recorded using an audio recorder to confirm informed voluntary participation. An independent community member acted as witness for voluntary informed decision making of participants to take part in the study. The study was approved by the Ethics Committee of the Faculty of Engineering and Natural Sciences, International Burch University.

### Development of the survey

The survey investigating awareness and attitudes toward *H. pylori* infection in physicians and the public population developed by Wu et al. was adapted to local characteristics of the general population in B&H (26). Initially, the survey underwent translation into Bosnian language and subsequent translation back into English. Before distributing the survey to the public, a pilot study was conducted among 20 non-health professionals, employees at the International Burch University, to ensure the clarity of the survey and its suitability for the general population. After the survey validation, it was published as a Google Form document, and widely distributed via the Internet (through social networks such as Facebook and Instagram, as well as calling and messaging applications such as Viber, WhatsApp, Messenger) across the entirety of B&H. It was open for 90 days and took ~15 min to complete it.

The questionnaire encompassed questions about knowledge and attitudes toward *H. pylori* infection. All participants were asked about their demographics, lifestyle habits associated with *H. pylori* infection, overall knowledge, and attitudes related to *H. pylori* testing. Categorization of the occupations was done according to the standard classification of occupations of the government of the Federation of B&H (27). Those who hadn't undergone *H. pylori* screening/testing were presented with questions about their reasons for not being tested and their willingness to undergo testing. For those expressing willingness, subsequent questions focused on their attitudes toward testing of their family members.

On the other hand, participants who had undergone *H. pylori* screening/testing were asked about their attitudes toward testing of their family members, test results, and treatment regimen. Participants who were infected but did not receive treatment were questioned about their reasons for abstaining from treatment. For patients who were infected and had received treatment, the survey investigated the experienced symptoms, treatment regimen employed, treatment duration, adverse drug reactions, follow-up attendance, symptoms' improvement post-treatment, and relapsed infection rate. The complete survey text can be made available upon request from the corresponding author.

### Statistical analysis

Data analysis was performed using MS Excel and R Statistical Software (v4.2.3; R Core Team 2021) (28). Participants fell into three knowledge groups: low (answering incorrectly or with “don't know”), moderate (1–3 correct answers), and high (4–5 correct answers) based on Wu et al.'s approach (26). The study linked *H. pylori* infection to participant demographics, habits associated with *H. pylori* infection, knowledge on *H. pylori*, screening status and status of *H. pylori* infection. It also explored symptoms' improvement post-treatment alongside adverse drug reactions and follow-up attendance. Using Fisher's exact test and Cramer's V measurement, associations were gauged as weak (<0.1), moderate (0.11–0.31), or strong (>0.31). Data were presented as numbers/percentages or median/IQR. In all statistical tests *p* < 0.05 was considered statistically significant.

## Results

### Demographic characteristics, habits, knowledge on *H. pylori*, screening status and status of *H. pylori* infection

Comprehensive demographic characteristics, lifestyle habits associated with *H. pylori* infection, knowledge on *H. pylori*, screening status and status of *H. pylori* infection of participants are presented in Table 1. A total of 1,031 people participated in the survey. Estimated participation rate was ~0.036%. Most of the included participants were in the youngest age group (18–29 years) (44.03%) while other age groups (30–39; 40–49; 50 and above) showed approximately similar percentage of includeness (18.62%, 16.88%, 20.47%, respectively). Most participants were women (73.04%), living in urban areas (65.37%), and highly educated (faculty 64.69%, secondary school 33.56%, elementary school 1.75%). Most participants in the survey were experts, scientists, technicians and other professional occupations (35.11%) as well as students (27.16%).

**Table 1:** Demographic characteristics, lifestyle habits associated with *H. pylori* infection, knowledge on *H. pylori*, screening status and status of *H. pylori* infection of participants (*N* = 1,031).

**Age**	***N***	**%**
18–29	454	44.03
30–39	192	18.62
40–49	174	16.88
50 and above	211	20.47
**Sex**		
Female	753	73.04
Male	278	26.96
**Educational level**		
Elementary school	18	1.75
Secondary school	346	33.56
Faculty	667	64.69
**Place of residence**		
Urban	674	65.37
Suburban	210	20.37
Rural	147	14.26
**Occupation**		
Agricultural, hunting and breeding, forest and fishing workers	12	1.16
Professional occupations (experts, scientists, technicians and other professional occupations)	362	35.11
Military occupations	13	1.26
Production workers (operators of machines, vehicles and assemblers of products, occupations for a non-industrial way of working in production)	22	2.13
Office and counter employees	103	9.99
Officials and members of legislative bodies, officials of state bodies, directors	30	2.91
Retiree (older than 65 years of age)	66	6.40
Service and commercial occupations	64	6.21
Simple occupations (elementary school education)	79	7.66
Student	280	27.16
**Lifestyle habits associated with** ***H. pylori*** **infection**		
Long-term^*^ coffee consumption	492	47.72
Long-term strong tea consumption	56	5.43
Long-term preserved food consumption	124	12.03
Long-term seafood consumption	66	6.40
Long-term sweets consumption	500	48.50
Long-term high-fat diet consumption	211	20.47
Long-term smoking	252	24.44
Long-term alcohol consumption	50	4.85
Long-term intake of carbohydrates	600	58.20
Other	24	2.33
None of the above	130	12.61
Participants with at least one aforementioned habits	860	83.41
**Additional habits associated with** ***H. pylori*** **infection**		
Chewing solid food for children	29	2.81
**Do you have a habit of dining alone?**		
Sometimes	749	72.65
Always	230	22.31
Never	52	5.04
**Participants heard of** ***H. pylori***		
Yes	818	79.34
No	213	20.66
**Participants screened/tested for** ***H. pylori*** **infection any time**		
**prior to survey**		
Yes	373	36.18
No	658	63.82
***H. pylori*** **infection any time prior to survey**		
Participants with *H. pylori* infection	246	65.95

^*^Lasting for more than a year.

Regarding personal habits associated with *H. pylori* infection, the largest number of participants, 83.41%, indicated at least one listed habit. In addition, most participants reported long-term (lasting for more than a year) consumption of carbohydrates (58.20%), sweets (48.50%) and coffee (47.72%).

Of all, 818 participants (79.34%) were familiar with *H. pylori* bacteria. Among the 373 (36.18%) participants that were screened/tested for *H. pylori* infection any time prior to survey, 246 (65.95%) reported their positive status.

### Effects of demographics, lifestyle habits, screening status and status of *H. pylori* infection on participants knowledge of *H. pylori*

Effects of demographic characteristics, lifestyle habits associated with *H. pylori* infection, screening status and status of *H. pylori* infection on participants knowledge of *H. pylori* are presented in Table 2. Sex, educational level, participants' screening for *H. pylori* status and occupation had significant effect on knowledge of *H. pylori*. Women compared to men (63.61% vs. 44.60%, *p* = 0.017), more educated participants compared to the less educated ones (faculty 60.72%, secondary school 55.78%, elementary school 27.78%, *p* = 0.000), those screened for *H. pylori* compared to the ones who were not screened (72.39% vs. 50.61%, *p* = 0.004), and those categorized as officials and members of legislative bodies, officials of state bodies, directors compared to production occupations (66.67% vs. 40.91% *p* = 0.001) showed significantly higher proportion of high knowledge of *H. pylori*.

**Table 2:** Effects of demographic characteristics, lifestyle habits associated with *H. pylori* infection, screening status and status of *H. pylori* infection on participants knowledge of *H. pylori* tested by Fisher's exact test with *p* < 0.05 considered statistically significant (shown in bold), and 4–6 correct options were referred to “High,” 1–3 correct options were referred to “Moderate,” and incorrect or unclear answers were referred to “Low.”

		**Low (0 correct)** ^*^		**Moderate (1–3 correct)**		**High (4–6 correct)**			
		***N***	**%**	***N***	**%**	***N***	**%**	**Total**	***p*** **-value**
	Overall	18	1.75	410	39.77	603	58.49	1,031	
Age	18–29	7	1.54	201	44.27	246	54.19	454	0.716
	30–39	2	1.04	65	33.85	125	65.10	192	
	40–49	5	2.87	68	39.08	101	58.05	174	
	50 and above	4	1.90	76	36.02	131	62.09	211	
Sex	Female	7	0.93	267	35.46	479	63.61	753	**0.017**
	Male	11	3.96	143	51.44	124	44.60	278	
Education level	Elementary school	0	0.00	13	72.22	5	27.78	18	**0.000**
	Secondary school	5	1.45	148	42.77	193	55.78	346	
	Faculty	13	1.95	249	37.33	405	60.72	667	
Residence	Urban	13	1.93	246	36.50	415	61.57	674	0.452
	Suburban	4	1.90	92	43.81	114	54.29	210	
	Rural	1	0.68	72	48.98	74	50.34	147	
Participants screened for *H. pylori* infection any time prior to survey	Yes	1	0.27	102	27.35	270	72.39	373	**0.004**
	No	17	2.58	308	46.81	333	50.61	658	
Occupation	Agricultural, hunting and breeding, forest and fishing workers	0	0.00	5	41.67	7	58.33	12	**0.001**
	Professional occupations (experts, scientists, technicians and other professional occupations)	9	2.49	116	32.04	237	65.47	362	
	Military occupations	0	0.00	7	53.85	6	46.15	13	
	Production workers (operators of machines, vehicles and assemblers of products, occupations for a non-industrial way of working in production)	0	0.00	13	59.09	9	40.91	22	
	Office and counter employees	3	2.91	38	36.89	62	60.19	103	
	Officials and members of legislative bodies, officials of state bodies, directors	0	0.00	10	33.33	20	66.67	30	
	Retiree (older than 65 years of age)	1	1.52	26	39.39	39	59.09	66	
	Service and commercial occupations	2	3.13	27	42.19	35	54.69	64	
	Simple occupations (elementary school education)	0	0.00	31	39.24	48	60.76	79	
	Student	3	107	137	48.93	140	50.00	280	

Military occupations and students had significantly lower frequency of high knowledge of *H. pylori* compared to other occupations (*p* = 0.001).

Age and residence had no significant effect on knowledge of *H. pylori*. Although participants aged 30–39 years and older participants aged 50 and up showed the highest frequency of high awareness regarding *H. pylori* (65.10%, 62.09%, respectively), compared to middle-aged (40–49 years) and younger adults (18–29 years) (58.05%, 54.19%, respectively) age had no significant effect on knowledge (*p* = 0.716). Similarly, although urban participants had a higher frequency of high knowledge answers (61.57%) compared to suburban and rural ones (54.29% vs. 50.34% respectively), residence did not significantly affect the knowledge (*p* = 0.452).

Overall, participants had a good knowledge of *H. pylori*, with more than a half (58.49%) answering four to six out of six questions correctly.

### Attitudes toward *H. pylori* screening/testing and treatment

Attitudes toward *H. pylori* screening/testing and treatment are presented in Table 3. Most of the participants (63.82%) were not screened for the *H. pylori* infection. The most common reason for not participating in screening was no obvious symptoms and not willing to check themselves (41.64%) and the test not being included in regular health examination (25.53%). Even though, not screened participants showed high frequency of support for screening (72.04%) while 25.23% were neutral and only 2.74% were against the screening.

**Table 3:** Attitudes toward *H. pylori* screening/testing and treatment.

**Participants screened/tested for *H. pylori* infection any time prior to survey**	***N***	**%**
Yes	373	36.18
No	658	63.82
**Questions for patients who WERE NOT screened for**		
***H. pylori***		
**Reason for not being screened**		
There are no obvious symptoms and I don't want to check them	274	41.64
It is not included in hospital physical examination	168	25.53
Other	132	20.06
I am young and not necessary to test it	79	12.01
I am old and worried about the risk of screening	5	0.76
**Support for** ***H. pylori*** **screening**		
Supporting screening	474	72.04
Neutral	166	25.23
Not supporting screening	18	2.74
**Questions for patients who WERE screened for** ***H. pylori*** **any**		
**time prior to survey**		
Which of the following methods would you choose to screen for *H. pylori*?		
Routine medical examinations when available	180	48.26
Go and test *H. pylori* on my own	178	47.72
Not clear	15	4.02
**If you are infected with** ***H. pylori*****, would you be advised**		
**by doctor to screen your family for** ***H. pylori*****?**		
Yes	175	46.92
No	134	35.92
Not sure	64	17.16
**What is your attitude toward national** ***H. pylori*** **screening?**		
Supporting screening	318	85.25
Neutral	46	12.33
Not supporting screening	9	2.41
**If you were infected with** ***H. pylori*****, would you take therapy to**		
**remove the bacteria from the body?**		
Yes	356	95.44
No	9	2.41
Not sure	8	2.14
**If you are negative for** ***H. pylori*****, but your family is positive**		
**for** ***H. pylori*****, would you recommend your family to take therapy**		
**to eliminate the bacteria from the body?**		
Yes	326	87.40
No	29	7.77
Not sure	18	4.83

The screened participants showed a higher level of support for screening programs, with 85.25% in favor. Among screened participants, 46.92% would recommend testing for other members of their family, whilst 35.92% would not. The majority would take the therapy to remove the bacteria (95.44%) and would recommend it to their family members who had tested positive (87.40%).

### *H. pylori* infection treatment regimen, adverse drug reactions and treatment effectivness

*H. pylori* infection treatment regimen, adverse drug reactions and treatment effectivness in participants who were tested positive at *H. pylori* screening/testing are presented in Table 4. Of 373 screened participans, 246 (65.95%) had confirmed *H. pylori* infection. Most of them, 231 (93.90%), received the treatment. While only minority of participants (15.38%) stated fear of relapse after infection and that the presence of *H. pylori* infection has no impact on health and life (30.77%) as specific reason for not receiving treatment, most participants stated no specific reasons for not receiving treatment (69.23%).

**Table 4:** *H. pylori* infection treatment regimen, adverse drug reactions and treatment effectivness in participants who were tested positive at *H. pylori* screening/testing.

**Participants with *H. pylori* infection**	***N***	**%**
Yes	246	65.95
No	127	12.32
**Participants with** ***H. pylori*** **infection who received treatment**		
Yes	231	93.90
No	15	6.10
**Reasons for not receiving treatment**		
Fear of relapse after eradication	2	15.38
Presence of *H. pylori* infection has no impact on health and life	4	30.77
Other	9	69.23
**Questions for participants who received treatment**		
Treatment regimen included antibiotic		
Yes	214	92.64
No	10	4.33
Not clear	7	3.03
**Received triple/quadruple treatment**		
Yes	213	92.21
No	11	4.76
Not clear	7	3.03
**Duration of the treatment**		
< 7 days	25	10.82
>14 days	70	30.30
11–14 days	49	21.21
7–10 days	57	24.68
Not sure	30	12.99
**Adverse drug reactions during treatment**		
Yes	134	58,01
No	97	41.99
**Reported adverse drug reactions** ^*^		
Abdominal pain	84	36.36
Diarrhea	42	18.18
Dry mouth	40	17.32
Constipation	20	8.66
Other	39	19.38
**Estimate of symptoms' improvement after the treatment**		
Improvement	88	38.10
Slight improvement	66	28.57
Complete improvement	44	19.05
No change	25	10.82
Worsening	8	3.46
**Re-testing after the treatment**		
Yes	172	74.46
No	53	22.94
Not sure	6	2.60
**Relapsed infection after the treatment**		
Yes	52	30.23
No	120	69.76
**Worry caused by treatment**		
Sometimes	66	28.57
All the time	43	18.61
Occasionally	42	18.18
Most of the time	42	18.18
Never	38	16.45
**I considered other treatment options due to unsatisfactory**		
**results of the prescribed therapy**		
Never	64	27.71
Sometimes	60	25.97
Occasionally	42	18.18
All the time	35	15.15
Most of the time	30	12.99

^*^Selection of more than one adverse drug reactions was possible.

Most of the treated participants were given antibiotics (92.64%) in a standard combination triple or quadruple therapy (92.21%), and the duration of the treatment in most participants lasted more than 14 days (30.30%). Of 231 treated participants, 58.01% experienced adverse drug reaction, mainly abdominal pain (36.36%) and diarrhea (18.18%). Also, 28.57% of participants experienced some level of worry caused by the treatment.

Most of the treated participants (85.72%) reported symptoms' improvement after therapy, with 28.57% reporting “slight improvement.” Eight participants (3.46%) said that the treatment worsened their symptoms. Some of the treated participants also continued to experience additional difficulties post-treatment, with 54 participants (23.38%) complained about having to give up some of their favorite foods due to illness, and 35 participants (15.15%) complained that illness had forced them to follow a diff8erent diet. The participants also reported issues such as belching or flatulence, stomach (upper belly) fullness, heartburn, and feeling nervous or afraid because of their condition. Other symptoms and difficulties experienced post treatment are shown in Table 5.

**Table 5:** Symptoms and difficulties experienced post-treatment.

**Do you still have the following symptoms after treatment?**	**Very often**		**Often**		**Sometimes**		**Almost never**		**Never**		**Did not answer**	
	***N***	**%**	***N***	**%**	***N***	**%**	***N***	**%**	***N***	**%**	***N***	**%**
Abdominal pain regardless of intensity	23	9.96	22	9.52	69	29.87	32	13.85	21	9.09	64	27.71
Stomach (upper abdomen) fullness	34	14.72	37	16.02	71	30.74	22	9.52	14	6.06	53	22.94
Belching or flatulence	44	19.05	47	20.35	65	28.14	20	8.66	13	5.63	42	18.18
Vomiting	12	5.19	13	5.63	32	13.85	23	9.96	69	29.87	82	35.50
Nausea	20	8.66	27	11.69	55	23.81	25	10.82	34	14.72	70	30.30
Heartburn	32	13.85	37	16.02	63	27.27	28	12.12	24	10.39	47	20.35
Bitter taste	15	6.49	23	9.96	48	20.78	25	10.82	47	20.35	73	31.60
Lack of appetite	12	5.19	21	9.09	45	19.48	29	12.55	45	19.48	79	34.20
You must give up eating some favorite food due to illness	54	23.38	26	11.26	50	21.65	19	8.23	30	12.99	52	22.51
Be dissatisfied with your life	19	8.23	24	10.39	38	16.45	27	11.69	54	23.38	69	29.87
Situation affecting the continuation of daily amateur activities	15	6.49	18	7.79	43	18.61	26	11.26	58	25.11	71	30.74
The relationship with your relatives and friends is affected due to illness	10	4.33	12	5.19	29	12.55	18	7.79	88	38.10	74	32.03
Restrictions on sexual life	4	1.73	10	4.33	32	13.85	18	7.79	87	37.66	80	34.63
Insomnia	19	8.23	28	12.12	43	18.61	22	9.52	54	23.38	65	28.14
Illness has forced you to adopt a separate diet	35	15.15	32	13.85	91	39.39	27	11.69	32	13.85	14	6.06
Feel sad because of your illness	20	8.66	16	6.93	39	16.88	26	11.26	60	25.97	70	30.30
Frustrated by your illness	21	9.09	17	7.36	30	12.99	28	12.12	60	25.97	75	32.47
Feel nervous or afraid due to illness (such as fear of canceration, etc.)	30	12.99	25	10.82	43	18.61	27	11.69	51	22.08	55	23.81

Most of the treated participants (74.46%) went to a re-testing after the treatment, with 30.23% of them who had relapsed infection after treatment.

### Effects of demographics, lifestyle habits, screening status and status of *H. pylori* infection on the incidence of *H. pylori* infection

The impact of demographic characteristics and lifestyle habits associated with *H. pylori* infection on the incidence of *H. pylori* infection is shown in Table 6. *H. pylori* infection was moderately associated with participants' age and occupation, whereas exhibited a strong association with lifestyle habits associated with *H. pylori* infection.

**Table 6:** The impact of demographic characteristics and lifestyle habits associated with *H. pylori* infection on the incidence of *H. pylori* infection.

	***H. pylori*** **infection**			
**Age (years)**	**Yes (%)**	**No (%)**	***p*** **-value**	**Cramer's V**
18–29	53.27	46.73	**0.003**	0.185
30–39	64.29	35.71		
40–49	71.91	28.09		
50 and above	76.34	23.66		
**Sex**				
Female	64.83	35.17	0.446	0.054
Male	69.88	30.12		
**Place of residence**				
Urban	64.84	35.16	0.282	0.092
Suburban	64.79	35.21		
Rural	73.91	26.09		
**Occupation**				
Professional occupations (experts, scientists, technicians and other professional occupations)	63.46	36.54	**0.000**	0.199
Simple occupations (elementary school education)	76.60	23.40		
Office and counter employees	63.04	36.96		
Student	62.79	37.21		
Service and commercial occupations	62.96	37.04		
Retiree (older than 65 years of age)	63.64	36.36		
Officials and members of legislative bodies, officials of state bodies, directors	78.57	21.43		
Agricultural, hunting and breeding, forest and fishing workers	80.00	20.00		
Military occupations	50.00	50.00		
Production workers (operators of machines, vehicles and assemblers of products, occupations for a non-industrial way of working in production)	77.78	22.22		
**Lifestyle habits associated with** ***H. pylori*** **infection**				
Long-term^*^ coffee consumption	22.36	77.64	**< 0.001**	0.353
Long-term strong tea consumption	21.43	78.57		
Long-term preserved food consumption	17.74	82.26		
Long-term seafood consumption	15.15	84.85		
Long-term sweets consumption	23.40	76.60		
Long-term high-fat diet	19.43	80.57		
Long-term smoking	26.19	73.81		
Long-term alcohol consumption	4.00	96.00		
Long-term intake of carbohydrates	25.67	74.33		
Other	70.83	29.17		
None of the above	23.08	76.92		

Results of the Fisher's exact test considered statistically significant for *p* < 0.05 (shown in bold). ^*^Lasting for more than a year.

The *H. pylori* infection rate was age-dependent with the oldest age group (50 and up) being the most (76.34%, *p* = 0.003), and the youngest age group being least likely to have been infected (46.73%). Agricultural, hunting and breeding, forest and fishing workers (80%) were most likely to have been infected, followed by officials and members of legislative bodies, officials of state bodies, directors (78.57%), as well as production workers (77.78%) and simple occupations (elementary school education) (76.60%) (*p* = 0.000). The highest proportions of non-infected participans were among military occupations (50.50%), students (37.21%) and participans with the secondary level of education i.e., service and commercial (37.04%) and office and counter workers (36.96%).

Among the lifestyle habits associated with infection, participants who reported long-term alcohol consumption were least likely to have been infected (4%, *p* < 0.001).

The sex and place of residence showed no impact on *H. pylori* infection rates.

### Effects of demographics, lifestyle habits, adverse drug reactions during treatment and re-testing after treatment on the estimated symptoms' improvement post-treatment

The impact of demographic characteristics, lifestyle habits associated with *H. pylori* infection, adverse drug reactions during treatment and re-testing after treatment on the estimated symptoms' improvement post-treatment is shown in Table 7. Estimated symptoms' improvement after the treatment was moderately associated with age, occupation, lifestyle habits associated with *H. pylori* infection and adverse drug reactions.

**Table 7:** The effect of demographic characteristics, lifestyle habits associated with *H. pylori* infection, adverse drug reactions during treatment and re-testing after treatment on the estimated symptoms' improvement post-treatment.

**Age (years)**	**Estimated symptoms' improvement post-treatment (%)**						
	**Improvement**	**Improvement**	**Complete improvement**	**No change**	**Worsening**	***p*** **-value**	**Cramer's V**
18–29	25.00	37.50	14.29	17.86	5.36	**2.00E-07**	**0.219**
30–39	60.87	19.57	13.04	4.35	2.17		
40–49	40.98	27.87	13.11	16.39	1.64		
50 and above	30.88	27.94	32.35	4.41	4.41		
**Sex**							
Female	40.23	29.89	18.39	9.20	2.30	0.189	**0.171**
Male	31.58	24.56	21.05	15.79	7.02		
**Place of residence**							
Urban	35.53	30.92	21.71	9.21	2.63	0.131	**0.146**
Suburban	42.22	20.00	13.33	17.78	6.67		
Rural	44.12	29.41	14.71	8.82	2.94		
**Occupation**							
Professional occupations	39.78	27.96	13.98	15.05	3.23	**1.00E-07**	**0.285**
Simple occupations (elementary school education)	44.12	20.59	23.53	11.76	0.00		
Student	23.08	38.46	19.23	11.54	7.69		
Office and counter employees	52.00	16.00	24.00	4.00	4.00		
Service and commercial occupations	47.06	29.41	17.65	5.88	0.00		
Retiree (older than 65 years of age)	30.77	38.46	30.77	0.00	0.00		
Officials and members of legislative bodies, officials of state bodies, directors	20.00	50.00	20.00	10.00	0.00		
Production workers	14.29	42.86	14.29	14.29	14.29		
Agricultural, hunting and breeding, forest and fishing workers	25.00	0.00	50.00	0.00	25.00		
Military occupations	50.00	50.00	0.00	0.00	0.00		
**Lifestyle habits associated with** ***H. pylori*** **infection**							
Long-term^*^ coffee consumption	33.96	28.30	22.64	9.43	5.66	**1.00E-07**	**0.232**
Long-term strong tea consumption	25.00	33.33	16.67	25.00	0.00		
Long-term preserved food consumption	31.82	27.27	9.09	22.73	9.09		
Long-term seafood consumption	37.50	12.50	12.50	25.00	12.50		
Long-term sweets consumption	37.27	28.18	19.09	13.64	1.82		
Long-term high-fat diet	35.90	28.21	7.69	20.51	7.69		
Long-term smoking	32.31	24.62	30.77	9.23	3.08		
Long-term alcohol consumption	50.00	0.00	50.00	0.00	0.00		
Long-term intake of carbohydrates consumption	32.87	33.57	18.18	11.89	3.50		
Other	18.75	43.75	18.75	6.25	12.50		
None of the above listed habits consumption	53.57	17.86	14.29	10.71	3.57		
**Adverse drug reactions during treatment**							
Yes	33.33	34.07	14.81	12.59	5.19	**0.023**	**0.236**
No	44.79	20.83	25.00	8.33	1.04		
**Re-testing after treatment**							
Yes	36.63	29.65	19.19	10.47	4.07	0.675	**0.110**
No	41.51	22.64	20.75	13.21	1.89		

Results of the Fisher's exact test considered statistically significant for *p* < 0.05 (shown in bold). ^*^Lasting for more than a year.

Youngest participants were least likely to report symptoms' improvement (25%, *p* = 2.00E-07) and most likely to report worsening of symptoms post-treatment (5.36%). Office and counter employees followed by military occupations were most likely to report improvement (52% and 50%, respectively), while agricultural, hunting and breeding, forest and fishing workers and retirees were most likely to report complete improvement (50% and 30.77%, respectively, *p* = 1.00E-07). Retirees (older than 65 years of age) and military occupations were least likely to report no change or worsening. The association was defined as moderate. Another moderate association was observed between consuming none of the listed habits associated with *H. pylori* infection and symptoms' improvement, i.e., participants who consumed none of the habits associated with *H. pylori* infection or reported long-term alcohol consumption were most likely to have reported symptoms' improvement (53.57%, 50.50%, *p* = 1.00E-07). Participants who reported long-term seafood consumption were most likely to report worsening (12.50%). Complete improvement and improvement were more likely for those with long-term alcohol consumption or no adverse drug reactions during treatment (*p* = 0.023).

No significant association was found between estimated symptoms' improvement after the treatment and sex, place of residence or re-testing after the treatment.

## Discussion

This was the first national survey on the knowledge and attitudes regarding diagnostics, treatment and recovery aspects related to *H. pylori* infection in the B&H population. The findings indicated that the general population had a good knowledge about *H. pylori* infection with more than half of the participants (58.49%) correctly answering nearly all questions. Of all participants, 36.18% underwent screening for *H. pylori*, with 65.95% testing positive, and of those, 93.90% received treatment, mainly antibiotics (92.64%) in a standard combination triple or quadruple therapy (92.21%) longer than 14 days (30.30%). Of all treated participants, 58.01% experienced adverse drug reaction, mainly abdominal pain (36.36%) and diarrhea (18.18%). Most of the treated participants, 85.72%, reported symptoms' improvement after therapy, but also went to a re-testing after the treatment (74.46%), with 30.23% of them who had relapsed infection. Being woman, highly educated, or already screened for *H. pylori* status was associated with higher knowledge of *H. pylori*, while being younger, student/with secondary level of education or with the history of long-term alcohol consumption was associated with lower rate of *H. pylori* infection. In addition, being older, with no history of consuming habits associated with *H. pylori* infection or with the history of long-term alcohol consumption was associated with symptoms improvement. Although most participants (63.82%) were not screened for the *H. pylori* infection, most of them (72.04%) as well as of those screened (85.25%) expressed support for a national *H. pylori* screening plan.

The relationships between higher educational levels and a lower prevalence of specific diseases demonstrate why knowledge is such an important aspect in both physical and psychological health. Notably, people with elementary school education or less had a disease prevalence rate 1.64 times greater than people with college degree or more (29). In this sense, our study yielded similar results, with more educated participants demonstrating greater knowledge than less educated persons. Furthermore, this study highlighted a potential link between knowledge levels and participants' inclination to participate in screening. Those who underwent screening for *H. pylori* infection demonstrated higher knowledge compared to those who did not undergo screening. This underscores the importance of educational programs for the general population, contributing to increased participation in screenings and disease awareness. The association with education is evident in professions such as officials and members of legislative bodies, state officials, directors, experts, scientists, technicians, and other professional occupations that require higher education. These occupations demonstrated higher knowledge levels. On the other hand, production workers, including machine operators, vehicle operators, and assemblers, as well as occupations that don't require higher education, exhibited lower knowledge levels.

Notably, the screening rate of 36.18% in our study surpasses the screening rate of 27.9% reported in a neighboring country Croatia (30). The predominant rationale behind the non-testing was the absence of symptoms. This poses a potential challenge, given that the infection typically manifests asymptomatically. Additionally, participants highlighted the omission of testing in routine hospital physical examinations. Conversely, those who underwent testing identified routine medical examinations, when accessible, as the optimal testing approach. Subsequently, it may be crucial to consider integrating *H. pylori* testing into the standard medical examinations within the healthcare system of B&H. This is also in line with strong support for national *H. pylori* screening shown in this study.

Study participants demonstrated strong endorsement for therapy, not only for themselves but also for their family members in the event of infection. This support is underscored by the noteworthy statistic that 93.90% of participants who tested positive for *H. pylori* underwent treatment. However, high resistance rates to clarithromycin (28.26%), quinolones (36.96%), and to both antibiotics (8.69%) were detected recently in the tested biopsies taken from the patients with dyspepsia in B&H (31). The reported clarithromycin resistance rate in B&H is slightly higher than in neighboring countries Serbia (24%) (32) and Croatia (21.2%) (33). This resistance could potentially limit the efficacy of the treatment, highlighting a potential rationale for considering its extension. This consideration is especially pertinent given the high relapsed infection rate among treated participants (30.23%).

Emphasizing the key role of extending treatment duration to increase eradication rates, a survey of the European *H. pylori* Care Registry found that physicians in the South-East region predominantly prescribed 7-day treatment regimens (34), as well as the triple treatment. Despite this, our results show that most treated participants were on treatment for longer than 14 days.

To consistently achieve eradication rates of 90% and mitigate the need for retreatment and patient dropout, physicians should be encouraged to employ quadruple therapies, as these regimens consistently yield eradication rates of ≥90% (34). This is in line with our results with nearly all participants reported receiving triple/quadruple treatment, and majority of them reported improvement after the treatment. Also, the authors of mentioned recent study on antibiotic resistance in B&H recommended the use of bismuth quadruple or non-bismuth concomitant quadruple therapy due to the high resistance rates to clarithromycin and quinolones (31). Accordingly, in the future it may be imperative to examine the effect of prolonged treatment as well as the effectiveness of the prescribed triple/quadruple treatment, especially in light of high rate of antibiotic resistance in B&H (31).

Assessing the association between demographic characteristics and lifestyle habits with *H. pylori* infectivity, numerous studies have identified poor hygienic conditions during the childhood, such as inadequate hand hygiene and insufficient access to clean water for washing, as well known risk factors associated with *H. pylori* infection (8–11, 35, 36), while some studies found no association (37, 38).

To date, no study on hygiene in B&H, nor on the association between hygiene and the prevalence of *H. pylori* was conducted.

In our study, we found that besides the youngest age group being least likely to have been infected (46.73%), participants who reported long-term alcohol consumption were, unexpectedly, also least likely to be infected (4%, *p* < 0.001). This is consistent with the results of a dose-response meta-analysis of observational studies that suggested moderate alcohol intake to be associated with a 22% reduction in *H. pylori* infection and its elimination (39). Additionally, a previous study by Kuepper-Nybelen et al. indicated an inverse relationship between alcohol consumption and *H. pylori* infection, suggesting that alcohol intake may contribute to the elimination of *H. pylori* infection among adults (40). This could serve as a foundation for further larger scale investigation to elucidate this association.

In addition, next to participants who consumed none of the habits associated with *H. pylori* infection, those who reported long-term alcohol consumption were also most likely to have reported symptoms improvement post-treatment (53.57%, 50.50%, *p* = 1.00E-07). Furthermore, symptoms improvement or complete resolution of symptoms were more likely for those with long-term alcohol consumption (50.00%, *p* = 1.00E-07). Our finding on potentially protective effect of long-term alcohol consumption is indeed intriguing and controversial. However, given the strong link between alcohol consumption and GC, the results of our study should only be used to further highlight the complexity of *H. pylori* infection and to suggest that more controlled studies are needed to determine whether alcohol consumption truly influences *H. pylori* prevalence or may facilitate symptoms' improvement post-treatment, and if so, through which mechanisms.

On the contrary, participants who reported long-term seafood consumption were not likely to have *H. pylori* infection, which is consistent with an earlier study demonstrated that a high intake of seafood was associated with a lower prevalence of *H. pylori* infection (41). However, participants who reported long-term seafood consumption were more likely to report worsening of symptoms (12.50%). It is surprising that factors such as seafood consumption, alongside with long-term conserved food intake, and those categorized as “other” demonstrated a negative impact on post-treatment recovery.

Notably, among the participants in this study, the younger ones were less likely to report improvement and more likely to report worsening. A plausible explanation for this pattern could be that younger people are less prone to experiencing dyspepsia or peptic ulcers, consequently reducing the likelihood of benefits in symptoms reduction from clearing the infection. This trend is consistent with the anticipated outcomes of screening and treatment in older participants (42).

Although our results provide background for more targeted studies, the study had several limitations. Firstly, the study included small convenience sample of participants and only those with sufficient digital literacy and Internet access which limits the generalizability of the findings. Secondly, the unequal sample of men and women as well the different age groups of participants could introduce bias. Also, the largest number of participants were highly educated which potentially might have caused the bias toward the higher knowledge score and more favorable attitudes toward screening and other healthcare interventions, which also limits the generalizability of the findings. Finally, no clear definitions for the time for reporting the screening/testing, treatment, re-testing for relapsed infection, and reporting the symptoms after the received treatment were given.

In perspective, it is crucial to assess the duration and the effectiveness of treatment for patients with *H. pylori* infection in B&H. It can partly be assessed by investigating physicians' awareness, knowledge and practices concerning *H. pylori* infection and its treatment. Also, considering the widespread population support for national *H. pylori* testing, the implementation of a national education campaign could prove beneficial, especially considering its absence thus far.

## Conclusions

This pioneering study offers a first comprehensive overview of knowledge, attitudes, diagnostics, treatment and recovery aspects toward *H. pylori* infection within the general population of B&H. Overall, B&H population demonstrated good knowledge toward *H. pylori* infection and expressed strong support for national *H. pylori* screening underscoring the importance of planning it in the public health initiatives in B&H. Also, due to the reported high relapsed infection rate, further effort needs to be directed toward community education, with a focus on the risk groups, i.e., older age groups, on effective measures for *H. pylori* prevention, early detection and treatment. Moreover, to gain a more comprehensive understanding of the *H. pylori* landscape in the B&H further research is needed to assess the duration and the effectiveness of treatment for patients with *H. pylori* infection.

## Data Availability

The original contributions presented in the study are included in the article/supplementary material, further inquiries can be directed to the corresponding author.
